# Detection of Pine Wilt Nematode from Drone Images Using UAV

**DOI:** 10.3390/s22134704

**Published:** 2022-06-22

**Authors:** Zhengzhi Sun, Mayire Ibrayim, Askar Hamdulla

**Affiliations:** School of Information Science and Engineering, Xinjiang University, Urumqi 830046, China; xjdxszz@163.com (Z.S.); mayire401@126.com (M.I.)

**Keywords:** UAV, deep learning, YOLO algorithm, pine wilt nematode

## Abstract

Pine wilt nematode disease is a devastating forest disease that spreads rapidly. Using drone remote sensing to monitor pine wilt nematode trees promptly is an effective way to control the spread of pine wilt nematode disease. In this study, the YOLOv4 algorithm was used to automatically identify abnormally discolored wilt from pine wilt nematode disease on UAV remote sensing images. Because the network structure of YOLOv4 is too complex, although the detection accuracy is high, the detection speed is relatively low. To solve this problem, the lightweight deep learning network MobileNetv2 is used to optimize the backbone feature extraction network. Furthermore, the YOLOv4 algorithm was improved by improving the backbone network part, adding CBAM attention, and adding the Inceptionv2 structure to reduce the number of model parameters and improve the accuracy and efficiency of identification. The speed and accuracy of the Faster R-CNN, YOLOv4, SSD, YOLOv5, and the improved MobileNetv2-YOLOv4 algorithm were compared, and the detection effects of the Faster R-CNN, YOLOv4, SSD, YOLOv5 and the improved MobileNetv2-YOLOv4 algorithm on trees with pine wilt nematode were analyzed. The experimental results show that the average precision of the improved MobileNetv2-YOLOv4 algorithm is 86.85%, the training time of each iteration cycle is 156 s, the parameter size is 39.23 MB, and the test time of a single image is 15 ms, which is better than Faster R-CNN, YOLOv4, and SSD, but comparable to YOLOv5. Compared with the advantages and disadvantages, comprehensively comparing these four indicators, the improved algorithm has a more balanced performance in the detection speed, the parameter size, and the average precision. The $F_{1}$ score of the improved algorithm (95.60%) was higher than that of Faster R-CNN (90.80%), YOLOv4 (94.56%), and SSD (92.14%), which met the monitoring requirements of pine wilt nematode trees. Faster R-CNN and SSD pine-wilt-nematode tree detection models are not ideal in practical applications. Compared with the YOLOv4 pine-wilt-nematode tree detection model, the improved MobileNetv2-YOLOv4 algorithm satisfies the condition of maintaining a lower model parameter quantity to obtain higher detection accuracy; therefore, it is more suitable for practical application scenarios of embedded devices. It can be used for the rapid detection of pine wilt nematode diseased trees.

## 1. Introduction

Pine wilt nematode is one of the most dangerous and devastating diseases in forest ecosystems in China and even in the world [1]. In recent years, the spread of pine wilt nematode has accelerated, and the damage has become increasingly serious. As of 2020, pine wilt nematode disease has occurred in 726 county-level administrative regions in 18 provinces in China [2]. (Pinus thunbergii) has expanded to Korean pine (Pinus koraiensis), larch (Larix gmelinii), and other species [3], and the epidemic directly threatens the security of nearly 60 million hm^2^ of pine forest resources in China. The monitoring of abnormally discolored wilt from pine wilt nematode disease is the key to the prevention and control of pine wilt nematode disease. Through monitoring, the disease situation can be grasped in time, and corresponding control work can be made accordingly, which is conducive to the management of pine wilt nematode disease.

Early monitoring methods were mainly based on ground surveys, that is, on-site inspections by forest rangers to determine whether an epidemic occurred in the forest area, preliminarily judge the degree of damage caused by the epidemic, and the location information of diseased trees, which is conducive to subsequent prevention and control. However, the use of artificial ground surveys will consume a lot of manpower and material resources, the efficiency is extremely low, and there is a time lag, especially in mountainous and remote areas with complex geographical environments, it is difficult to fully understand the dynamics of the pine needle nematode epidemic promptly, and it is easy to miss the best preventive measures. It is a good time, which leads to the further spread of the epidemic.

In 1994, my country adopted the U.S. Forest Service aerial photography system to monitor the epidemic. Since then, domestic researchers have begun to use a large amount of space remote sensing technology to monitor pests and epidemic areas. Compared with ground measurements, aerospace remote sensing technology can save manpower and improve monitoring efficiency. However, due to the limitation of spatial resolution of aerial remote sensing images, only large-scale epidemic monitoring can be performed, and it is difficult to monitor individual affected trees. Rainy weather affects the clarity of remote sensing images and creates many difficulties for aerial photography. The long flight cycle of satellites is in stark contrast to the characteristics of short infection time and fast spread of pine trees, which is likely to cause monitoring gaps.

Since their inception, UAVs have mainly used in the military field [4]. With the development and application of UAV technology, UAV technology has been widely used in crop protection, photography, disaster relief, and other civilian fields. Researchers at home and abroad have used UAV remote sensing technology to monitor trees affected by pine forest diseases. First, the affected trees were found from high-resolution drone images of pine forests using a visual judgment method, which is subjective. Tao Huan et al. [5] used the HSV threshold method to identify diseased trees in the obtained UAV images, and the overall accuracy of the method was (60% to 65%). Wang Hong et al. [6] used portable ground equipment to collect the spectral reflectance of the pine trees with the diseased beetle and established the spectral characteristics of the diseased pine trees, which achieved an accuracy rate of more than 80% in the prediction of the diseased pine trees. Liu Xialing et al. [7] obtained high-resolution images and used a multi-template detection method to identify trees affected by pine forest diseases in different disease stages. The results show that this method can effectively improve the detection efficiency of diseased trees compared with visual interpretation. Therefore, machine learning methods have also adopted by related researchers. Hu Gensheng et al. [8] used a dual-spectral camera installed on a UAV platform to obtain images of pine forests and then used a weighted support vector data description algorithm to identify diseased trees. Aimed at the problem that the traditional aerial photo identification method cannot quickly locate the pest outbreak center and track the spread of the disaster, Sun Yu et al. [9] proposed a real-time monitoring method based on deep learning. The depthwise separable convolutional network as a feature extractor achieves an average precision of 97.22% during testing. Zhang Sulan et al. [10] used the spectral reflectance of Masson pine as the data source to analyze the ridge trace of 14 spectral characteristic parameters and constructed a pine wilt nematode disease ridge regression monitoring model. The test results showed that the determined coefficient R^2^ of the constructed pine wilt nematode ridge regression model was 0.8686, the mean square error RMSE was 0.2735, and the average estimation accuracy was 87.15%, which provides technical support for the early monitoring and control of pine wilt nematode disease. The method of combining Fast R-CNN and UAV remote sensing proposed by Huang Huayi et al. [11] has an accuracy rate of 90%. For UAV image data, Song Yining et al. [12] used a linear spectral clustering superpixel algorithm to monitor and locate diseased trees and used support vector machines to accurately locate diseased trees. The test results show that in the monitoring results of the linear spectral clustering superpixel method, the intersection ratio with manual detection results was greater than 58%. Lu Mingzhan and others have explored the rapid identification technology of abnormal trees by drones based on artificial intelligence. Tests have proved that the accuracy of this technology in identifying diseased trees is more than 90%, which is 70 times faster than manual speed. Liu Jincang et al. [13] used UAV remote sensing technology to take color images of forest areas and used the CRF algorithm to classify and identify diseased pine trees according to the color characteristics and texture characteristics of ground objects in the images. After experimental verification, this method has a good effect on the monitoring of pine wilt nematode disease. Based on the analysis of pine wilt nematode disease prevention and control work in Chongqing, Wu Honggan et al. [14] further carried out the remote sensing monitoring of dead pine trees by unmanned aerial vehicles and gave detailed working steps, which provided a basis for the research on the spatiotemporal regularity of pine wilt nematode disease.

It was used in Canada in 1988 to detect leaf moth disease. Joon-Bum et al. [15] used IKONOS satellite imagery to monitor epidemic areas in South Korea. With the continuous development of satellite remote sensing technology, foreign researchers began to use high-resolution satellite remote sensing data and hyperspectral satellite remote sensing data to monitor trees with pine wilt nematode disease. After obtaining high-quality remote sensing images by UAV, Mutiara et al. [16] used an artificial neural network and support vector machine to classify and detect trees affected by the disease; Iordachel et al. [17] used a random forest method to classify the found diseased trees. Pham et al. [18] realized that the use of empirical methods and artificial intelligence to analyze and predict vegetation parameters in hyperspectral remote sensing is flawed and proposed a new deep learning model to solve the problem of class imbalance and gradient normalization. Testing the model achieves a class-balanced accuracy of 78.32%. S.Natesan et al. [19,20,21,22,23] proposed a new method of UAV monitoring tree species based on residual neural network, using the images collected by UAV in the past three years to train the artificial neural network, respectively conducted two groups of experiments, obtained 80% and 51% tree species classification accuracy. Franklin et al. proposed a new method for UAV tree species classification based on object image analysis and machine learning, using image segmentation technology to segment the acquired images. During the experiment, after using the random forest algorithm for classification, the overall correctness of the independent verification samples was obtained. The rate reached 78%. Kim MJ et al. [24] used drones to collect orthophoto images of 423 pine trees suspected of being infected with pine wilt nematode disease in six areas and used a satellite navigation system to conduct field investigations. The study found that the occurrence of pine wilt nematode infection has nothing to do with tree species, which improved monitoring and work efficiency in pine wilt nematode endemic areas. In their research, Onishi et al. used a commercial drone to obtain aerial images of forests and performed image segmentation operations to separate a single tree canopy. Using an open-source deep learning framework, a machine vision system for automatic tree classification was constructed. The classification accuracy of seven tree species in the environment reached 89.0%, providing a cost-effective tree classification tool for forest researchers and managers. Kestur et al. [25,26,27] used UAV as a new type of remote sensing platform applied in the field of ecology, proposed an ELM spectral space classification method for monitoring, delineation, and counting carried out spectral space classification of tree crowns in RGB images and proved that the performance of this algorithm is better than K-Means spectral space clustering method. Lees et al. [28] used drones to collect high-resolution images in pine wilt nematode endemic areas and used artificial neural network ANN and support vector machine technology to monitor pine trees that were killed and withered by pine wilt nematode disease. Using satellite images and deep learning-based image segmentation algorithms in Guatemala forests, Wyniawskyj et al. [29,30] realized an automated model for forest area detection at the pixel level of satellite images.

All the above methods can realize the monitoring of diseased trees, but the use of UAV remote sensing to monitor pine wilt nematode trees is the general trend.

## 2. The Location of the Study Area

From 2017 to 2019, Yantai City, Shandong Province, carried out the census and disposal of pine wilt nematode trees. Among them, the Queen’s Mountain Forest Area in Penglai District, Yantai City, is the main epidemic area. After the pine tree is infected, it gradually begins to die from the top of the tree. During manual inspections during this period, an early-infected pine was discovered. Through in-depth investigation, the diseased pine trees in the forest area were found one after another, and the disease has spread in the forest area. Taking Queen Mountain and its surrounding areas in the Penglai District, Yantai City, as the study area, we explored a new method for detecting diseased trees.

**Figure 1:**
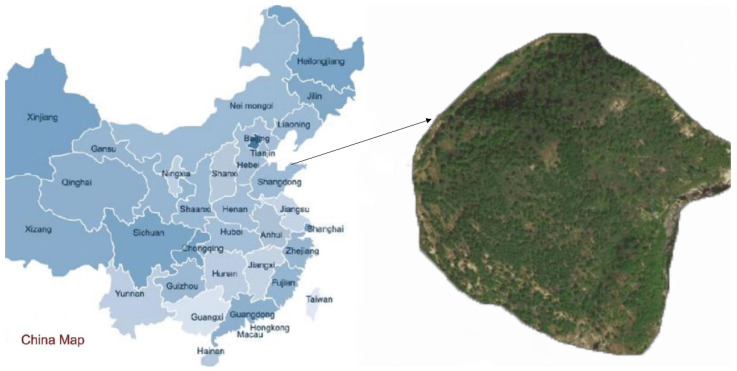
Location information of the study area.

The location of the study area is shown in Figure 1. During the Xianfeng and Tongzhi years of the Qing Dynasty, there was war in the Jiaodong area. At that time, many villages were in crisis, and they built walls in the mountains to protect themselves. Queen’s Mountain is also called “Weizi Mountain”, which is also related to this. At that time, it was a woman with strong martial arts who led the surrounding villages to practice and defend the mountain. Under her leadership, the mountain people repelled the enemy’s attack many times, but in the battle, she was unfortunately hit by a dark arrow and died in battle. To commemorate her, the locals named “Weizi Mountain” “Queen’s Mountain”, which has a long history. There are 34 species of ancient and famous trees in the forest area, with a total of tens of thousands of trees.

## 3. Creation of Experimental Datasets

This chapter takes pine wilt nematode diseased trees as the research object and uses drone aerial photography technology to collect images of diseased trees in Queens Mountain Forest Area, Penglai District, Yantai City. At a flight altitude of 100 m above sea level, the M600 drone carried out aerial photography missions to the pine forest in the Queen’s Mountain Forest area according to the route planned in advance. The Yu-2 UAVs performed shooting tasks at average altitudes of 50 m, 100 m, 250 m, and 300 m.

### 3.1. UAV Parameters

**Figure 2:**
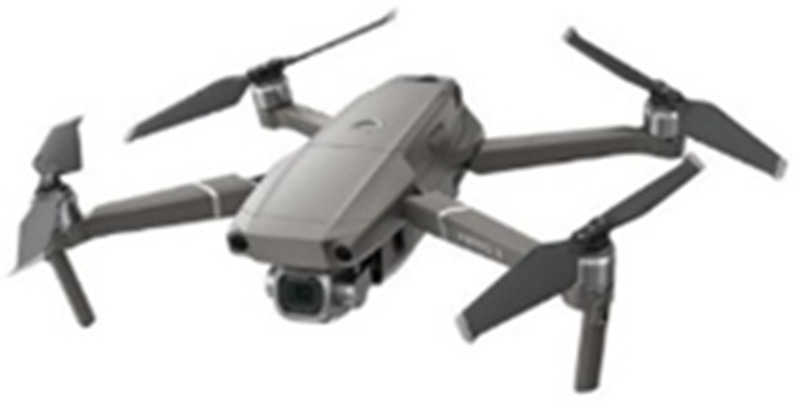
DJI “Yu” series MAVIC2 aircraft.

**Figure 3:**
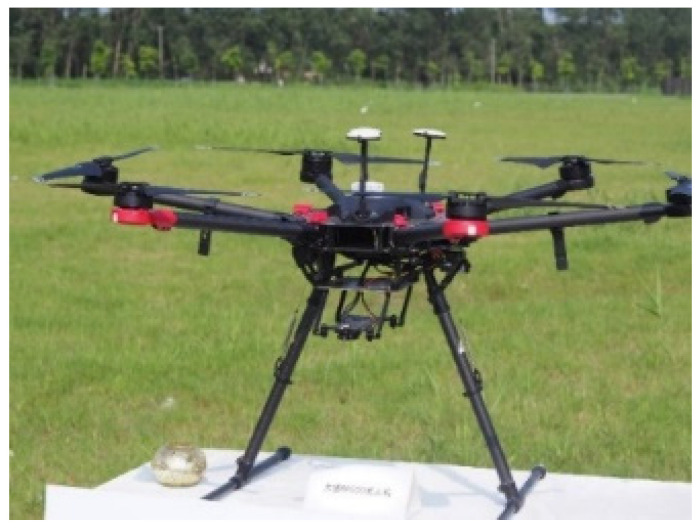
DJI M600 aircraft.

In this paper, the pine wilt nematode diseased trees were taken as the research object, and the RGB image information of the pine wilt nematode trees in the Queens Mountain forest area of Penglai District, Yantai City, China, was collected by using the image acquisition method of aerial photography of the forest area by the UAV airborne camera. The equipment and its parameters used to collect images from aerial photography are shown in Figure 2 and Figure 3 and Table 1.

### 3.2. Cut Aerial Images

Quality checks are performed on each image, and invalid images are removed. The identification of pine wilt nematode trees requires high color saturation and brightness, but due to the influence of impurities in the air, the original image color brightness is not clear. The original image was processed by using the dark channel dehazing method. The dehazing process can saturate the color of the image and improve the brightness, which is beneficial to the identification of pine wilt nematode trees. Because the images obtained by UAV aerial photography technology are too large, each image must be more than 4 G, and some images can even be as high as 12 G. The image size is too large. If the original image is downsampled, the available features will be reduced. To better obtain the feature information of the image, the image after the dehazing process is cropped and divided, the frame size is 500 × 500, and the unit is the pixel.

**Figure 4:**
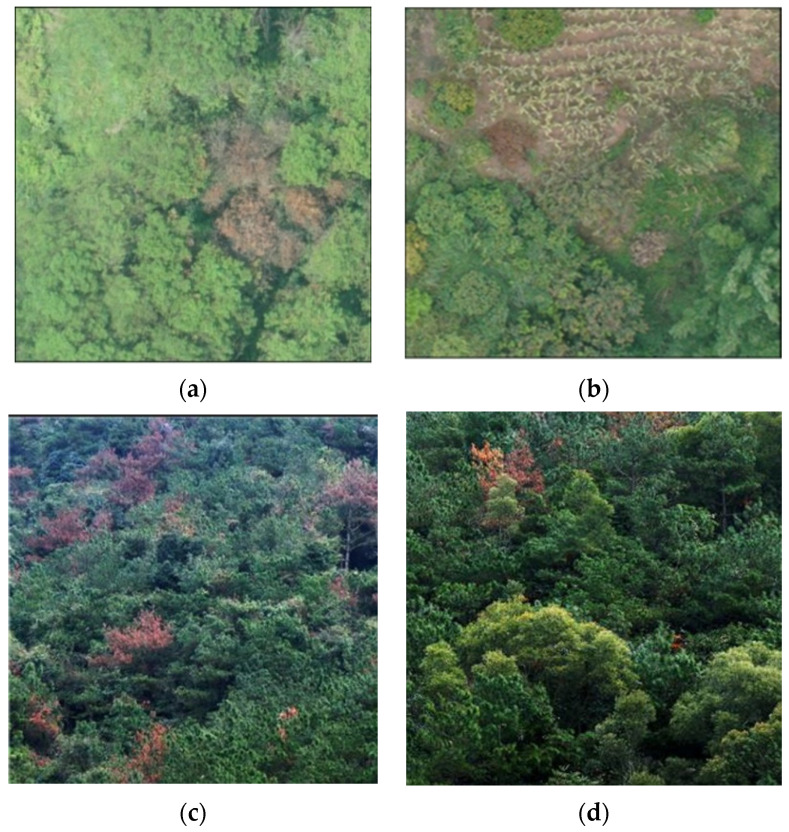
Collected images of pine wilt nematode trees. (**a**) DJI M600 of Figure 1; (**b**) DJI M600 of Figure 2; (**c**) DJI Royal 2 of Figure 1; (**d**) DJI Royal 2 of Figure 2.

I created a total of 116,012 image samples of forest areas. The amount of image data is shown in Table 2:

The pine trees with pine wilt nematode disease photographed by the two are shown in Figure 4. Figure 4a,b were taken by the M600, and Figure 4c,d were taken by the Royal 2 drone.

### 3.3. Manually Labeled Datasets

**Figure 5:**
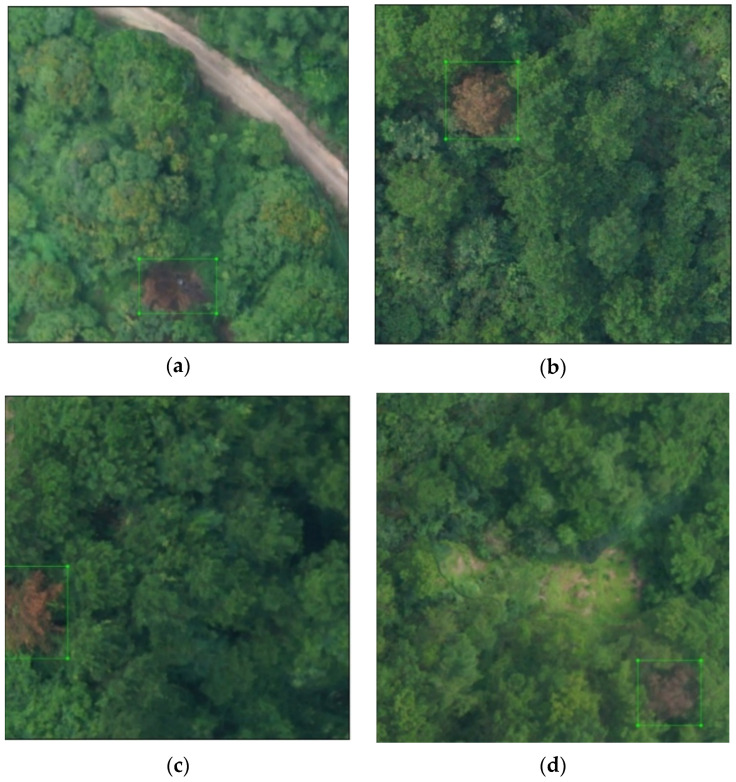
Annotated dataset example. (**a**) Annotation example 1; (**b**) Annotation example 2; (**c**) Annotation example 3; (**d**) Annotation example 4.

In the Queen’s Mountain Forest, Penglai District, Yantai City, after drone aerial photography was used to collect images of pine trees, the Labellmg professional image labeling software was used to manually label the collected 116,012 images and transform them into the PascalVOC type. Click the mouse to use the green box to select the object and enter the name of the object on the label. In the figure, the tree with pine wilt nematode disease is labeled, and the label is “illtree”, and the labeling result is shown in Figure 5.

**Figure 6:**
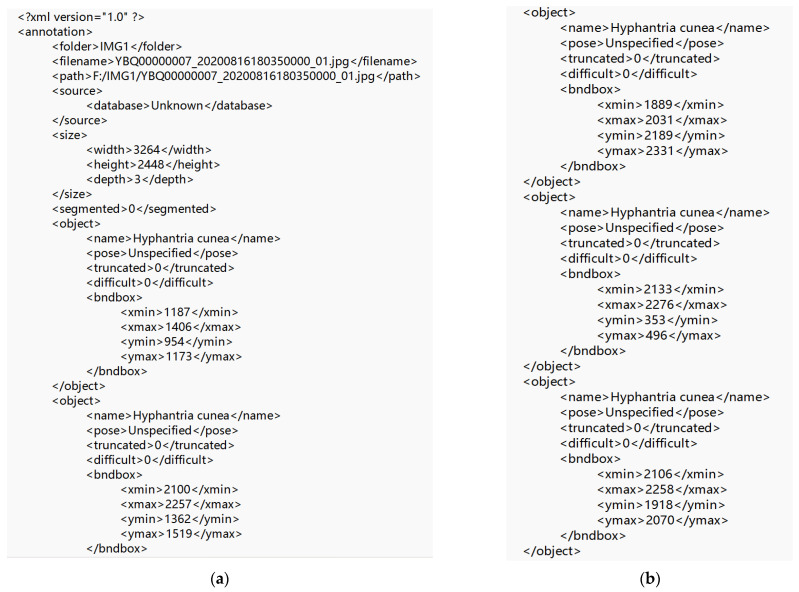
XML file tag information. (**a**) XML file tag information 1; (**b**) XML file tag information 2.

All of the label files were obtained after manual labeling. The XML label file contained the category information of the labeled object and the coordinate information of the labeling box of the diseased tree, that is, the bounding box of the object, as shown in Figure 6. This was marked in the format of [xmin, ymin, xmax, and ymax], xmin and xmax are the coordinates of the upper left corner of the object frame, ymin, ymax are the coordinates of the lower right corner of the object frame; how many objects need to be identified in a picture? How many objects are there in its XML file?

**Figure 7:**
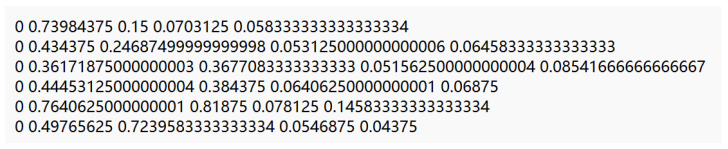
Annotated information.

After the image was successfully annotated with the annotation software, the annotation information of the dataset was read, and the annotation information in the label file was read out using a python script and stored in the txt file, and all of the images appeared in the labels folder: label information. As shown in Figure 7, the coordinate information of the bounding box of the object in the XML tag file was read.

### 3.4. Classification of Datasets

Finally, the images containing trees with pine wilt nematode disease were selected in the cut image as a sample set for the detection of pine wilt nematode trees. The dataset was divided into three categories: training, testing, and validation. The default training, testing, and validation follow the 8:1:1 ratio for random classification.

## 4. Introduction to the YOLOv4 Model

**Figure 8:**
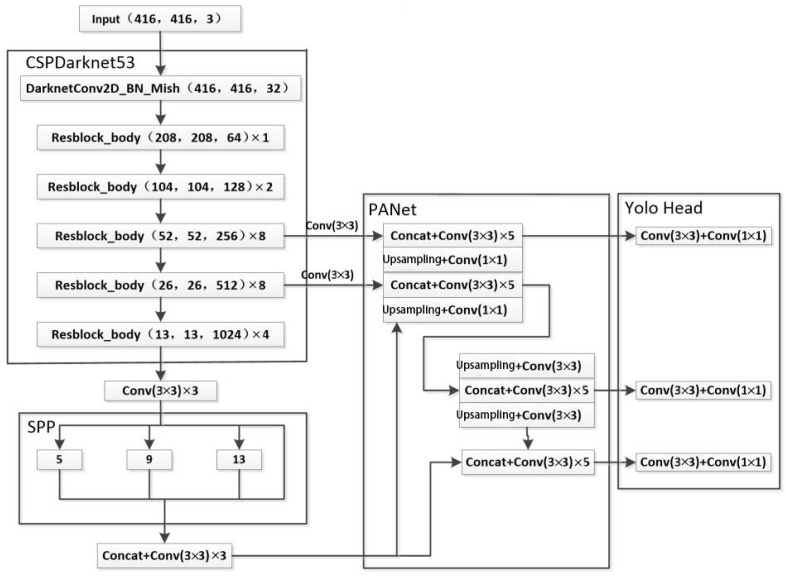
Network model structure figure of YOLOv4.

The YOLOv4 algorithm uses three feature layers for classification and regression prediction, mainly composed of three parts: the backbone feature extraction network CSPDarknet53, the enhanced feature extraction network SPP + PANet, and the prediction network YOLO Head. The specific network model structure is shown in Figure 8.

First, extract features from the input image through the backbone network CSPDarkNet53. In this process, use a convolutional layer with a convolution kernel size of 3 × 3 and a stride of two to downsample the input five times in a turn to form three effective feature layers 13 × 13, 26 × 26, and 52 × 52.

In addition, the 13 × 13 feature layer is fused with multi-scale receptive fields through spatial pyramid pooling networks (SPPnet), and the fused 13 × 13 feature layer is combined with the 26 × 26 generated by the backbone network, 52 × 52 feature layers together through the feature fusion network PANet to fuse the shallow layer with sufficient detailed features and the deep layer with rich semantic features, which improves the problem of poor detection of small objects. In this process, a total of two upsampling, two downsamplings, multiple convolutions, and concatenation operations.

Finally, the feature layer obtained after the feature fusion network is sent to the detection network, and the input image is divided into 13 × 13, 26 × 26, and 52 × 52 grid images by classification and regression, and the large, medium, and small three are detected respectively. Objects of different scales. Each grid is responsible for predicting three bounding boxes; each bounding box predicts the location information of the object (including the center coordinates and width and height of the predicted box) and the confidence of the existence of the object if there are k categories in the dataset, the final output feature. The number of channels on the graph is 3 × (5 + k). Compared with the two-stage object detection algorithm, the YOLOv4 algorithm not only improves the detection accuracy but also speeds up the detection speed.

## 5. Improved MobileNetv2-YOLOv4 Model

The objection detection algorithm based on deep learning is one of the important branches of computer vision. The objection detection is based on the convolutional neural network to complete the image analysis and processing to solve the problem of “what is the objection object? Where is the objection object?” Point out the category of all interested objection objects in the image and the location of the objection object in the image, which is the classification and positioning of the objection object. At present, objection detection algorithms are mainly divided into two categories: one-stage and two-stage. The one-stage includes SSD and YOLO, and the two-stage includes R-CNN, Fast R-CNN, and Faster R-CNN. The YOLO algorithm has been used on objects such as tomatoes, boats, birds, and planes. Compared with the two-stage algorithm, the YOLO algorithm has a faster detection speed. The YOLO detection algorithm has made great progress, but due to the large proportion of equipment resources in the convolutional neural network, it is not suitable to run directly on mobile and embedded devices. To reduce the number of model parameters, maintain the same detection accuracy and further improve the precision measurement speed, the use of a lightweight deep learning network optimizes the backbone feature extraction network. Among them, the MobileNet series is a typical representative of lightweight networks. The MobileNetv2 network is a structure generated by neural architecture search technology based on MobileNetv2. It is mainly improved in the following three aspects: (1) Improve the backbone network; (2) Add CBAM attention; (3) Add the Inceptionv2 structure.

### 5.1. Ideas for Improvement

(1) Improve the backbone network

Since the YOLOv4 algorithm uses CSPDarknet53 as the backbone network, although it can extract effective feature information, the network structure is quite complex, resulting in an excessive number of parameters, which is not ideal in terms of practicability. Therefore, this chapter uses MobileNetv2 as the backbone network in the YOLOv4 algorithm, which can reduce the number of parameters while ensuring its accuracy and form the MobileNetv2-YOLOv4 model. The MobileNetv2-YOLOv4 model extracts three effective feature layers through Mobileetv2, which are 52 × 52, 26 × 26, and 13 × 13, respectively. Since the detailed information in the 13 × 13 feature map is gradually lost in the process of feature extraction, convolution with a kernel size of 3 × 3 and a stride of four is used on the 52 × 52 feature for downsampling with the 13 × 13 feature. Graphs are fused to form bottom-up connections.

(2) Add CBAM attention

**Figure 9:**
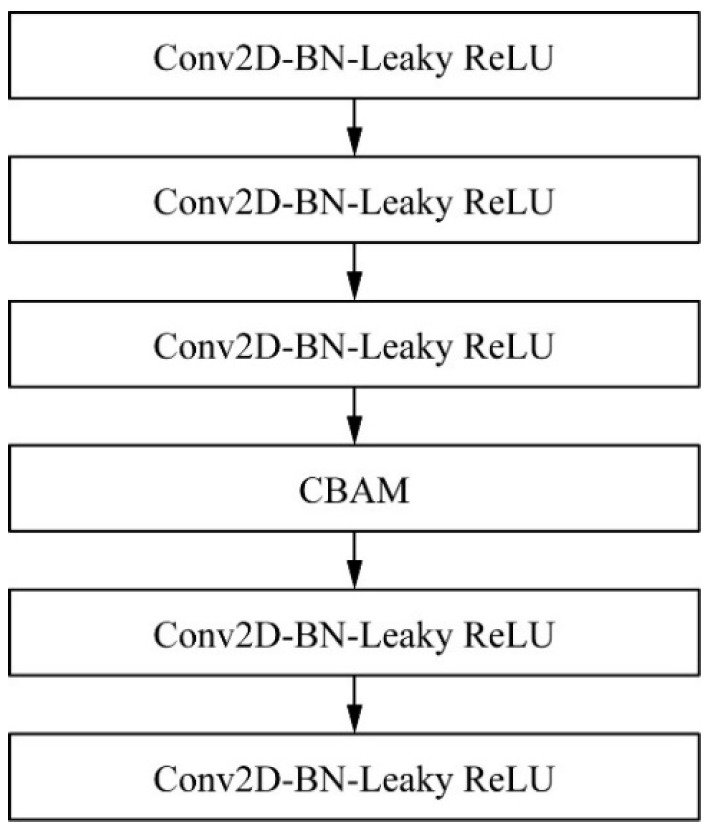
CBC module figure.

The feature fusion network of the YOLOv4 algorithm is located after the backbone network, and the three effective feature layers extracted from the backbone network are further convolutionally fused to obtain more representative features. In this process, since the features have the same expressive power in the two dimensions of the feature map channel and space, the extracted features are redundant, which makes the detection effect of the model worse. Therefore, the CBAM attention mechanism is added to the five convolutions after the feature fusion network of YOLOv4 is spliced to build the CBC module. In this way, the network can focus on more important features in the training process, ignore redundant features, and improve detection accuracy. The CBC module is shown in Figure 9.

(3) Add Inceptionv2 structure

**Figure 10:**
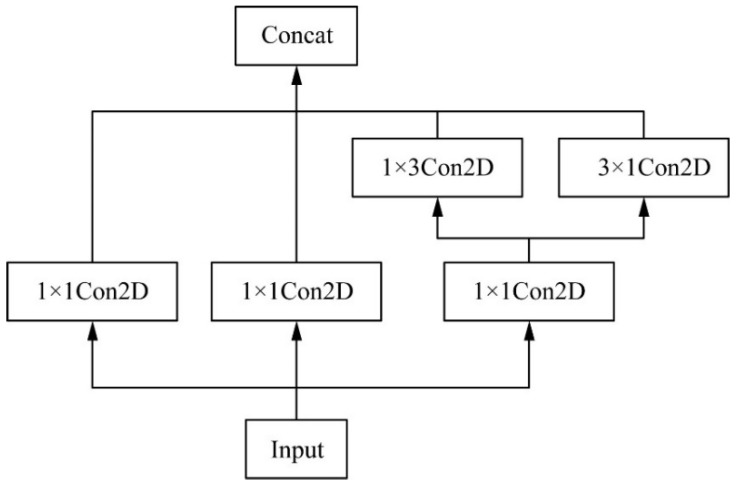
Inception 3 × 3 structure figure.

Since the detection network of the YOLOv4 algorithm only uses the 3 × 3 convolution kernel to integrate the feature map, the integrated feature information is weak, and the integration process requires a large number of parameters. If the one-dimensional convolution of 1 × 3 and 3 × 1 is replaced by 3 × 3, the convolution can increase the nonlinear expression of the model while reducing the number of parameters. Drawing on the structure of the Inceptionv 2 model, the 3 × 3 convolution in the last layer of the YOLOv4 algorithm is changed to the Inception 3 × 3 structure, and an ICP module is constructed: first, use three parallel 1 × 1 convolutions to reduce the number of channels, and then replace the original 3 × 3 convolution with a 1 × 3 convolution and 3 × 1 convolution, and then add it to one of the 1 × 1 convolutions, and finally fuse it, which reduces the number of parameters while deepening the depth of the network and improving the performance of the network. The structure of Inception 3 × 3 is shown in Figure 10.

### 5.2. Improved MobileNetv2-YOLOv4 Network Structure

**Figure 11:**
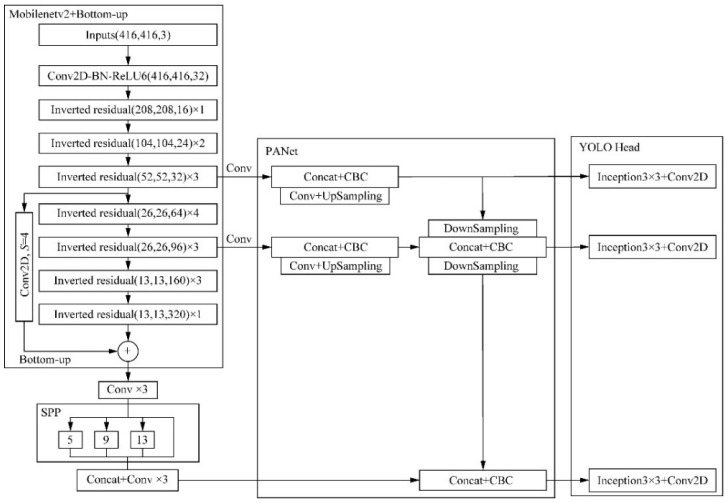
MobileNetv2-YOLOv4 network structure figure.

The overall network structure Figure of the improved algorithm can be obtained from the improvement points, as shown in Figure 11.

**Figure 12:**
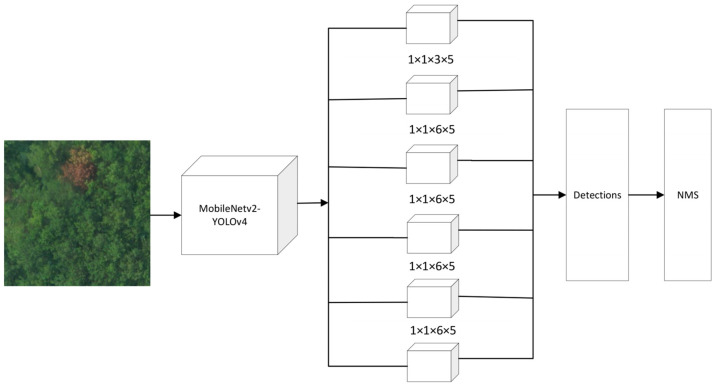
MobileNetv2-YOLOv4 detection process.

Figure 12 shows the detection process of MobileNetv2-YOLOv4’s pine-wilt-nematode tree detection model. The input image is first passed through the MobileNetv2-YOLOv4 feature extractor. In the process of generating the feature map, the detection boxes are divided into six scales for regression training, and finally, the categories and bounding boxes are obtained through the non-maximum suppression operation. Table 3 shows the overall structure of MobileNetv2-YOLOv4, which mainly includes one initial convolution and 19 bottleneck blocks.

## 6. Experimental Part

### 6.1. Experimental Environment

The YOLOv4 object detection methods used in this paper are based on the TensorFlow deep learning open-source framework. The hardware and software configurations of the GPU nodes used are shown in Table 4.

The hardware and the software configuration of the mobile workstation are shown in Table 5.

### 6.2. Experimental Parameters

The parameter settings of this experiment are shown in Table 6.

The other command-line parameter settings required to perform this experiment are shown in Table 7.

### 6.3. Evaluation Metrics and Methods

In practical application scenarios, a single indicator cannot meet the requirements, it is not suitable, and it is not easy to judge the quality of a model. This paper improves the YOLOv4 algorithm, but whether the improvement can stand the test is a question. Therefore, this paper uses the Accuracy Precision (AP), training time, parameter size, and test time to evaluate the performance of the improved algorithm. The concepts are as follows:

1. Average precision refers to the mean of the accuracy under different recall rates, which is the average precision in the test set.
2. Training time refers to the time required to train an iterative cycle.
3. Test time refers to the time required to detect a single image.

In addition, when evaluating the test accuracy of the improved algorithm, the precision rate (Precision, P), recall rate (Recall, R), and $F_{1}$ score are used.

(1) $$\mathrm{Precision}=\frac{TP}{TP+FP}$$

In Formula (1), TP+FP is the total number of detection frames calibrated by the detection model on the pine wilt nematode disease tree test set.

(2) $$\mathrm{Recall}=\frac{TP}{TP+FN}$$

In Formula (2), TP+FN is the total number of detection frames calibrated by the model on the test set.

(3) $$\mathrm{Accuracy}=\frac{TP+TN}{TP+FP+TN+FN}$$

Formula (3) represents the accuracy of the model, where TP, FP, TN, and FN have specific meanings shown in Table 8.

$F_{1}$ score is an indicator used in statistics to measure the accuracy of a binary classification model. It also takes into account the precision rate and recall rate of the classification model and refers to the harmonic mean of the precision rate and recall rate, as shown in Formula (4).

(4) $$F_{1}=2\cdot \frac{\mathrm{Precision}\cdot \mathrm{Recall}}{\mathrm{Precision}+\mathrm{Recall}}$$

The training platform was fixed, and the experimental environment was guaranteed to remain unchanged. Using the same image data, the three object detection algorithms were based on the improved MobileNetv2-YOLOv4 algorithm, the Faster R-CNN algorithm, the SSD algorithm, the YOLOv4 algorithm, and the YOLOv5 algorithm. These five algorithms detected the same image and finally obtained the detected data results.

According to the results, analysis and study of the performance of the five algorithms, comparing the average accuracy, training time, parameter size, and the test time of the five algorithms, and judging the improvements to the MobileNetv2-YOLOv4 algorithm, Faster R-CNN algorithm, SSD algorithm, YOLOv4 algorithm, and YOLOv5 algorithm, it was determined that they have a better performance for detecting pine wilt nematode trees. In addition, by using the algorithm’s precision rate, recall rate, and $F_{1}$ score and other indicators, we analyzed and compared the different performances of the Faster R-CNN algorithm, SSD algorithm, YOLOv4 algorithm, YOLOv5 algorithm, and improved MobileNetv2-YOLOv4 algorithm.

### 6.4. Experimental Results and Analysis

During the model training process, after the MobileNetv2-YOLOv4 pine-wilt-nematode tree-detection model was trained 250 times, the mAP curve peaked at 0.6. The model converged after 250 training runs, so the 300 training results were used.

**Figure 13:**
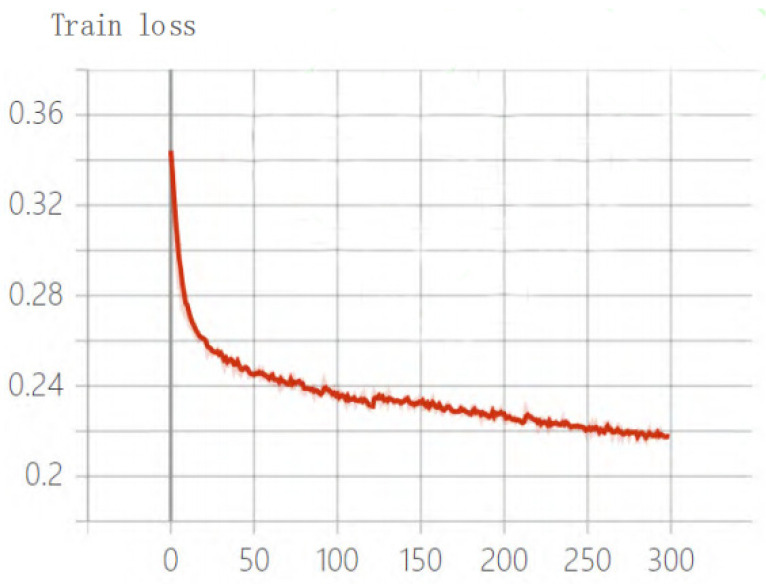
Training loss of the improved algorithm.

The total training loss of the improved algorithm is shown in Figure 13. After training for 300 epochs, the loss function tends to be stable, and the variation range is small, indicating that the learning rate is set reasonably, the loss value was finally stabilized at 0.21 and 0.22, and the training was finally in a state of convergence.

(1) Performance comparison of different algorithms

Different algorithms were selected, and the results of the identifying images were also different. As shown in Table 9, this paper used five algorithms to compare the data of the victim wilt detection. In terms of average precision, the improved algorithm has an average accuracy of 86.85%, which is 7.21%, 6.29%, 3.37%, and 3.73% higher than that of the Faster R-CNN, SSD, YOLOv4, and YOLOv5 algorithms, respectively; in terms of training time, after the algorithm is improved, the training time of each iteration cycle is 156 s, which is 168 s, 131 s, and 93 s less than the Faster R-CNN, SSD, and YOLOv4 algorithms, respectively, but 33 s more than YOLOv5. In terms of parameter size, after the algorithm is improved, the model parameter size is 39.23 MB, which is 412.95 MB, 330.19 MB, and 174.69 MB smaller than Faster R-CNN, SSD, and YOLOv4 algorithms, but 16.27 MB larger than YOLOv5. During the test time, a single image was improved by the algorithm. In the test, the time is 15 ms, which is 67 ms, 32 ms, and 6 ms less than the Faster R-CNN, SSD, and YOLOv4 algorithms, respectively, but 8 ms more than YOLOv5.

In terms of average precision, training time, parameter size, and test time, the improved algorithm performs better than Faster R-CNN, SSD, and YOLOv4 algorithms; in terms of training time, test time, and model parameter size, YOLOv5 is better than Faster R-CNN, SSD and the improved algorithm performed excellently, but the average accuracy of Faster R-CNN, SSD, and YOLOv5 algorithm tests was not as good as that of the improved algorithm. In the task of pine-wilt-nematode-disease tree detection, the accuracy requirements were higher, so the improved algorithm was better than YOLOv5. The improved algorithm can obtain higher detection accuracy under the condition of maintaining a lower model parameter quantity, and the improved algorithm is more suitable for practical application scenarios of embedded devices. Therefore, a comprehensive comparison is made on the four evaluation indicators of average precision, training time, parameter size, and test time, and the improved MobileNetv2-YOLOv4 algorithm has better detection performance. At present, in terms of detection accuracy, the detection performance of the improved YOLOv4 algorithm still needs to be improved, but it meets the detection requirements of pine wilt nematode trees and can be applied to the detection of pine wilt nematode trees in different regions.

(2) Performance Evaluation of Improved Algorithms

**Figure 14:**
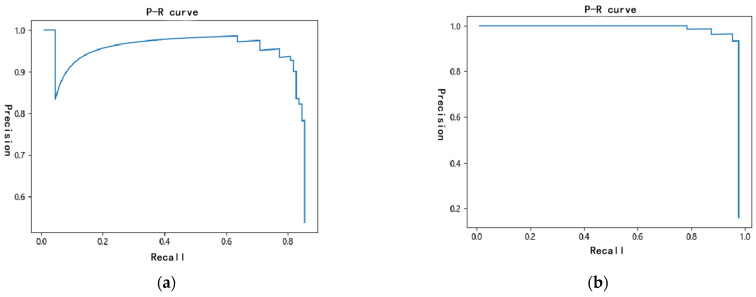
P-R curve. (**a**) YOLOv4; (**b**) MobileNetv2-YOLOv4.

In the performance indicators of the object detection model, Recall and Precision represent recall rate and precision rate, respectively, and precision rate and recall rate are a pair of contradictory measures. Generally speaking, when the precision rate is high, the recall rate is low, and when the recall rate is high, the precision rate is low. In this experiment, the PR curves before and after the improvement of the identification of trees with pine wilt nematode disease are shown in Figure 14. As can be seen from Figure 14a,b, the P-R curve of the latter can completely cover the P-R curve of the former, and the performance of the latter is better than that of the former; that is, the improved algorithm has a better performance.

The important performance index of the pine-wilt-nematode tree detection model is to reduce the omission of the pine-wilt-nematode tree detection during the detection process. As shown in Table 10, using the Faster R-CNN pine-wilt-nematode tree detection model, among the 95 objections, the number of false positives was three, the number of false negatives was 13, the accuracy rate reached 83.16%, and the recall rate reached 85.87%, the $F_{1}$ score reached 90.80%; the SSD pine-wilt-nematode-disease tree detection model, among 95 objections, the number of false positives was one, the number of false negatives was 13, the accuracy rate reached 85.26%, and the recall rate reached 86.17% the $F_{1}$ score reached 92.14%; using the YOLOv4 pine-wilt-nematode tree detection model, 10 of the 87 objections were underreported, the accuracy rate reached 89.69%, the recall rate reached 89.69%, and the $F_{1}$ score was 94.51%. Using the improved MobileNetv2-YOLOv4 pine-wilt-nematode tree detection model, 8 out of 87 objects were underreported, the model accuracy rate reached 91.58%, the recall rate reached 91.58%, and the $F_{1}$ score was 95.60%. From the analysis of the experimental results, it can be seen that the number of false positives for the YOLOv4 and the improved MobileNetv2-YOLOv4 pine-wilt-nematode tree detection model for the objection is 0, and the number of false positives was lower than that of Faster R-CNN and SSD pine-wilt-nematode-tree detection model. Therefore, the application effect of the Faster R-CNN and SSD pine wilt nematode tree detection model is not ideal in actual scenarios, and the model parameters are also very large, so they are not suitable for running on mobile and embedded devices.

The analysis of the experimental results shows that the accuracy of the improved algorithm is 8.42%, 5.41%, and 1.89% higher than that of the Faster R-CNN, SSD, and YOLOv4 algorithms, respectively, indicating that after the algorithm is improved, the detection accuracy is better. The recall rate of the improved algorithm is 5.71%, 5.41%, and 1.89% higher than that of Faster R-CNN, SSD, and YOLOv4 algorithms, respectively, indicating that after the algorithm is improved, the detection in the test area is more comprehensive. The $F_{1}$ scores of the improved algorithm are 4.80%, 3.46%, and 1.04% higher than those of Faster R-CNN, SSD, and YOLOv4 algorithms, respectively, which can meet the needs of pine wilt nematode epidemic control.

### 6.5. Algorithm Performance Test

**Figure 15:**
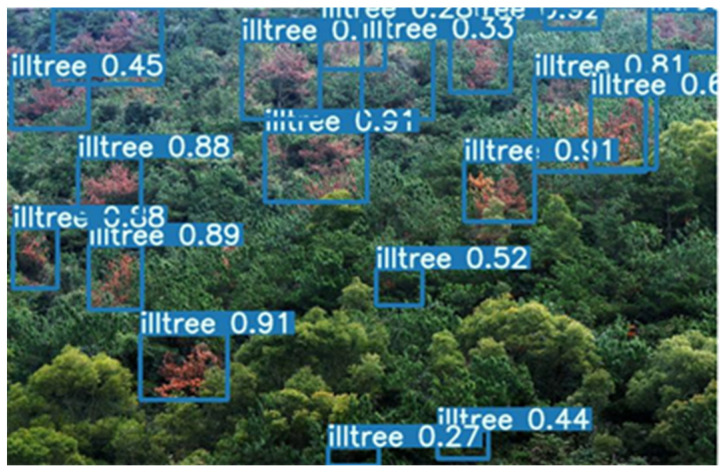
The figure of the detection effect of pine wilt nematode trees.

After the training is completed, the detection effect of the model on the test set is shown in Figure 15.

**Figure 16:**
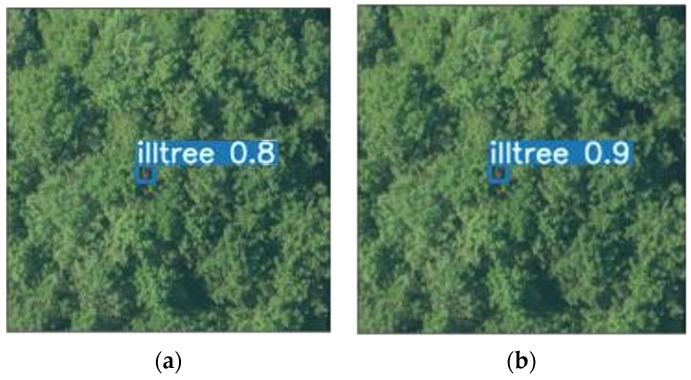
Algorithm comparison figure. (**a**) Algorithm detection results of YOLOv4; (**b**) Algorithm detection results of MobileNetv2-YOLOv4.

After the algorithm is improved, especially when the object is small, the accuracy rate has improved. As shown in Figure 16, for the same object, using MobileNetv2-YOLOv4, the accuracy rate reaches 0.9, which is 1% higher than YOLOv4.

**Figure 17:**
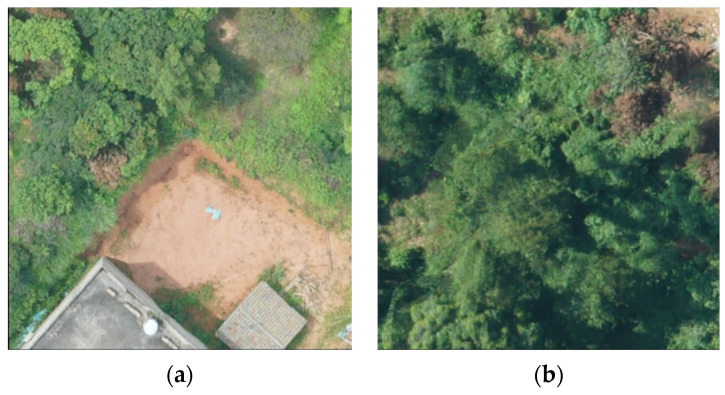
The picture of a diseased tree similar to a ground feature. (**a**) Figure 1; (**b**) Figure 2.

**Figure 18:**
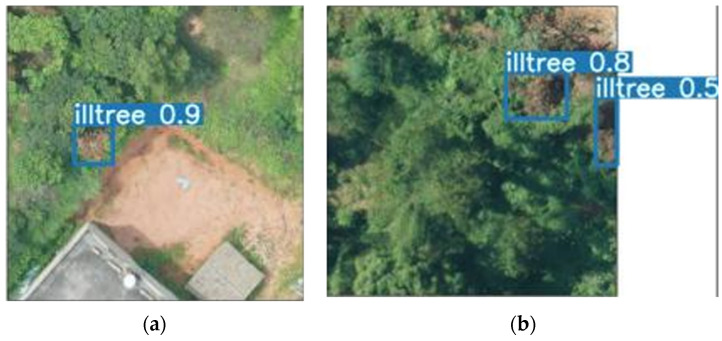
The picture of MobileNetv2-YOLOv4 detection. (**a**) Figure 1; (**b**) p 2.

As shown in Figure 17, in both images, there are trees with pine wilt nematode disease and ground objects with similar colors to the trees with pine wilt nematode disease. In the first image, the diseased tree has a similar color to the land next to it. In the second image, the same is true. In this regard, using the MobileNetv2-YOLOv4 model, the diseased tree can also be successfully detected, and a relatively good accuracy rate can be obtained, as shown in Figure 18.

### 6.6. Analysis of Factors Affecting Accuracy

When analyzing the detection results, it shows that the improved MobileNetv2-YOLOv4 algorithm can better complete the task of automatic identification of pine wilt nematode diseased trees and can achieve better detection accuracy. However, in this process, it will also cause missed detections and misjudgments. The main factors that affect the detection accuracy are as follows:

1. Similar features. There are features similar to abnormally discolored wilt in the test area. Because the image features of similar ground objects and abnormally discolored wilt are similar, and the color and texture are close, it is easy to cause misjudgment of abnormally discolored wilt during identification, thus affecting the accuracy of identification.
2. Stand canopy closure. Statistical results show that the forests with high canopy closure have overlapping crowns, which will cause partial or complete occlusion. The partially occluded canopy of discolored trees has incomplete shapes and changes in shape characteristics, which are prone to missed detections during identification, and those that are completely occluded cannot be identified.
3. Slope aspect. The shaded slope area lacks sunlight and has shadows. The brightness of the canopy image in the shaded area is low, resulting in the dark color of the abnormally discolored tree canopy, and the image color and texture characteristics are not obvious, and it is easy to miss detection during identification.
4. Resolution. Due to the consistent flying height of the drone, the undulation of the terrain will cause changes in the size of the resolution. The higher the terrain, the higher the resolution, the larger and clearer the canopy, the lower the terrain, the lower the resolution, and the smaller the canopy features of abnormally discolored wilt will occur; and it is easy to miss detection during identification.

### 6.7. Discussion

In the monitoring of pine wilt nematode trees, UAV remote sensing technology has low cost, wide range, high flexibility, and high efficiency and has been well applied in the automatic identification of pine wilt nematode trees [28,29]. In this study, the improved MobileNetv2-YOLOv4 algorithm was used to identify trees with pine wilt nematode disease automatically, and the $F_{1}$ score reached 95.60%, which met the needs of pine wilt nematode disease prevention and control. Compared with the overall accuracy (60–65%) of the HSV thresholding method proposed by Tao Huan et al. [30]. The accuracy is significantly improved. The detection accuracy of this study is slightly higher than the accuracy (90%) of the method combining Fast R-CNN and UAV remote sensing proposed by Huang Huayi et al. However, this study considered other factors for the detection of pine wilt nematode trees. Therefore, the reliability of the accuracy is higher.

In this study, the YOLOv4 algorithm was improved according to the fast-spreading speed of pine wilt nematode. The simplified object detection framework can speed up the detection speed. The test time of the improved MobileNetv2-YOLOv4 algorithm for a single image is 15 ms, which is 67 ms, 32 ms, and 6 ms less than Faster R-CNN, SSD, and YOLOv4, respectively. When the number of detections is too large, the time spent on image detection by the improved MobileNetv2-YOLOv4 algorithm will be much less than that of Faster R-CNN, SSD, and YOLOv4, which can meet a wide range of requirements and monitoring needs. The improved MobileNetv2-YOLOv4 algorithm reduces the number of parameters and the size of the model, reduces the requirements for hardware equipment for the identification program, and improves the efficiency of identification. It can realize real-time drone monitoring of pine wilt nematode trees, grasp the situation of diseases and insect pests in forest areas in real-time, and track the epidemic situation. The development of pine wilt nematode meets the demand for timeliness of pine wilt nematode control.

The limitations of inverted residual block: feature maps encoded by the intermediate expansion layer should be first projected to low-dimensional ones, which may not preserve enough useful information due to channel compression. Replace ReLU with a linear activation function. The inverted residual with the linear bottleneck. The module first expands the input low-dimensional compressed representation to high dimensions, using lightweight depthwise convolutions for filtering, then projects the features back to the low-dimensional compressed representation using a linear bottleneck. A shortcut was introduced, and the last ReLU was removed and changed to Linear. When the step size is one, the 1 × 1 convolution is first performed to increase the dimension, then the depthwise convolution is performed to extract features and then the dimension is reduced by the linear point-by-point convolution. Add the input and output to form a residual structure. When the step size is two, the shortcut structure is not added because the size of the input and output do not match, and the rest are the same.

The color change of abnormally discolored wilt from pine wilt nematode disease is a dynamic process, and the color of abnormally discolored wilt from pine wilt nematode disease is different in different stages. The color of the needles of the pine trees infected with pine wilt nematode did not change significantly in the early stage, but in the middle stage, the crown color was yellow-brown, and in the later stage, the crown color was reddish-brown or brown. The method proposed in this paper can only automatically identify the abnormally discolored wilt in the middle and late stages and cannot effectively identify the pine wilt nematode diseased wilt with no obvious color change in the early stage, nor can it distinguish the abnormally discolored wilt caused by pine wilt nematode disease from other diseases or natural environment stress.

Use hyperspectral data that contains more information. The camera used in this paper to collect data contains three bands of visible light, RGB, and the amount of information contained in the image is limited. Because the process of pine disease infection is dynamic, the changes in its external shape in different disease stages have certain differences. The method in this paper can identify the pine trees in the middle and late stages of the disease according to the color and texture characteristics, and it is difficult to detect the pine trees in the early stage of the disease. Monitor the outbreak comprehensively. In addition to the changes in appearance, the spectral values of pine trees will also change after being infected, and the spectral characteristics of reflectance at different disease stages are also different. Hyperspectral technology uses many narrow electromagnetic bands to obtain continuous spectral information of ground objects, so hyperspectral data can be used as the basis for detecting pine trees at different disease stages. In addition, the bands that can best reflect the information changes of diseased trees are selected from the hyperspectral images through band selection to improve the detection accuracy of diseased trees. The diseased pine trees can be detected comprehensively by combining hyperspectral data and deep learning methods.

We aimed to improve the detection methods for pine-wilt-nematode-diseased trees. The pine-wilt-nematode-disease detection method mentioned in this paper is based on deep learning. The object detection model needs a lot of matrix operations and floating-point operations in the process of training and detection and needs to use a supercomputing platform with powerful computing power to complete. The corresponding operation increases the application cost. To reduce computing resource requirements, you can try a more advanced object detection model architecture or use new pine-wilt-nematode tree-identification methods such as spectral data analysis to reduce computing resource requirements and computing power costs while ensuring the Detection performance.

## 7. Conclusions

In this study, UAV remote sensing technology was used to obtain ultra-high spatial resolution pine images, and the improved MobileNetv2-YOLOv4 algorithm was used to identify abnormally discolored trees from pine wilt nematode disease. The main conclusions are as follows:

1. Using the improved MobileNetv2-YOLOv4 algorithm to identify abnormally discolored wilt from pine wilt nematode disease, the average precision reached 86.85%, the training time for each iteration cycle was 156 s, the parameter size was 39.23 MB, and the test time for a single image was 15 ms. Faster R-CNN, SSD, and YOLOv4 are inferior to the improved algorithm in terms of average precision, training time, parameter size, and test time. YOLOv5 outperforms the improved algorithm in training and detection time, but the average precision is lower than the improved algorithm, so compared to Faster R-CNN, SSD, YOLOv4, and YOLOv5, the improved algorithm is more balanced in the model parameters, the detection speed, and the detection accuracy, with better performance. Comprehensively comparing the average precision, training time, parameter size, and test time, the improved MobileNetv2-YOLOv4 algorithm has better performance.
2. In the pine wilt nematode tree detection task, a comprehensive comparison is made on the four evaluation indicators of average precision, training time, parameter size, and test time. The detection accuracy of wilt nematode trees is low, the number of objections missed and false positives is large, the number of model parameters is large, the detection speed is slow, and the actual application effect is not ideal, and it is not suitable for running on mobile and embedded devices. Compared with the YOLOv4 model, the MobileNetv2-YOLOv4 model has improved significantly in terms of average accuracy, detection accuracy, and detection speed, and the training time and model parameters are reduced, and the performance is improved. Compared with the YOLOv5 model, the parameter quantity of the improved model is slightly higher than that of YOLOv5, but the average accuracy is greater. For a comprehensive comparison, the MobileNetv2-YOLOv4 model can obtain higher detection accuracy under the condition of lower model parameter quantity, and the improved algorithm is more suitable for practical application scenarios of embedded devices.
3. Similar features, stand canopy closure, slope aspect, and terrain will lead to insufficient feature acquisition or wrong feature detection during automatic identification, resulting in missed detection, which will affect the accuracy, but the impact is small.

## Figures and Tables

**Table 1:** UAV and airborne camera parameters.

UAV, Camera	Parameter	Value
DJI “Yu” series of MAVIC aircraft	Maximum take-off mass/g	1100
	Dimensions (L × W × H)/mm	322 × 242 × 84
	Maximum flight altitude/m	500
	Maximum flight speed/(km·h^−1^)	50
	Max flight time/min	31
DJIFC200 camera	Dimensions (L × W × H)/mm	Gimbal, lens, and body
	Photo format	JPEG
	Image Sensor	1/2.3″ CMOS
	Photo resolution	4000 × 3000
DJI M600 aircraft	Maximum take-off mass/g	15,500
	Dimensions (L × W × H)/mm	1668 × 1518 × 727
	Maximum flight altitude/m	300
	Maximum flight speed/(km·h^−1^)	65
	Max flight time/min	32

**Table 2:** Segmented aerial forest image.

Sample	Quantity
Forest image	116,012

**Table 3:** MobileNetv2-YOLOv4 structure.

Input	Operator	t	c	n	s
224^2^ × 3	conv2d	-	32	1	2
112^2^ × 32	bottleneck	1	16	1	1
112^2^ × 16	bottleneck	6	24	2	2
56^2^ × 24	bottleneck	6	32	3	2
28^2^ × 32	bottleneck	6	64	4	2
14^2^ × 64	bottleneck	6	96	3	1
14^2^ × 96	bottleneck	6	160	3	2
7^2^ × 160	bottleneck	6	320	1	1
7^2^ × 320	conv2d 1 × 1	-	1280	1	1
7^2^ × 1280	Avgpool 7 × 7	-	-	1	-
1^2^ × 1280	conv2d 1 × 1	-	k	-	

Note: Each line describes a sequence of 1 or more identical (modulo stride) layers, repeated *n* times. All layers in the same sequence have the same number *c* of output channels. The first layer of each sequence has a stride *s,* and all others use stride 1. All spatial convolutions use 3 × 3 kernels. The expansion factor t is always applied to the input size.

**Table 4:** GPU node hardware and software details.

Project	Configure
CPU	Intel Xeon E5 v5
RAM	128 GB DDR5
Graphics processing unit	NVIDIA Tesla K80 × 2
Operating system	Linux (RedHat 6.9)
Deep Learning Framework	TensorFlow 2.3.1
CUDA	CUDA 10.1

**Table 5:** Mobile workstation hardware and software details.

Project	Configure
CPU	Intel i7-11700K
RAM	16 GB DDR4
Graphics processing unit	NVIDIA GeForce RTX 3060
Operating system	Linux (Ubuntu 18.04)
Deep learning framework	Tensorflow 2.3.1
CUDA	CUDA 10.1

**Table 6:** Experimental parameter settings.

Parameter	Project
Learning rate	0.01
Learning rate momentum	0.873
Weight decay	0.005
The maximum number of iterations	300
Non-maximum suppression	0.6
Confidence	0.1
Intersection over union	0.5

**Table 7:** Command-line parameter settings.

Command-Line Arguments	Parameter Meaning	Setting Value
Batch	Unified input image scale	16
Epochs	The number of times the dataset participated in training	300
Device	Training equipment	GPU
Weights	Weight file	MobileNetv2-YOLOv4.pt

**Table 8:** Specific meaning.

	P (Positive, 1)	N (Negative, 0)
T (True, 1)	TP (Positive samples predicted by the model to be positive)	TN (Negative samples predicted by the model to be negative)
F (False, 0)	FP (Negative samples predicted by the model to be positive)	FN (Positive samples predicted by the model to be negative)

**Table 9:** Performance comparison of different object detection models.

Models for Object Detection	Backbone	Average Precision%	Training Time/s	Parameter Size	Testing Time
Faster R-CNN	VGG 16	79.64	324	452.18	82
SSD	VGG 16	80.56	287	369.42	47
YOLOv4	CSPDarknet 53	84.48	249	213.92	21
YOLOv5	YOLOv5 s	84.12	123	22.96	7
This paper	MobileNetv2-YOLOv4	86.85	156	39.23	15

Note: The average precision is the average precision of the testing set, the training time is the time required to train one epoch, and the testing time is the time required to recognize a single image.

**Table 10:** Performance comparison of different object detection models.

	TP	TN	FP	FN	Precision	Accuracy	Recall	$F_{1}\;\mathrm{Score}$
Faster R-CNN	79	0	3	13	96.34%	83.16%	85.87%	90.80%
YOLOv4	85	0	0	10	100%	89.69%	89.69%	94.56%
SSD	81	0	1	13	98.78%	85.26%	86.17%	92.14%
This paper	87	0	0	8	100%	91.58%	91.58%	95.60%

Note: *TP*, *FP*, and *FN* indicate the quantity of true positive, false positive, and false negative, respectively. *P*, *R*, and $F_{1}$ indicate precision, recall, and $F_{1}$ score.
